# Green Wearable Sensors and Antennas for Bio-Medicine, Green Internet of Things, Energy Harvesting, and Communication Systems

**DOI:** 10.3390/s24175459

**Published:** 2024-08-23

**Authors:** Albert Sabban

**Affiliations:** Department of Electrical Engineering, ORT Braude College, Karmiel 2161002, Israel; sabban@braude.ac.il

**Keywords:** wearable sensors, green electronics, renewable energy, energy harvesting, healthcare applications, IoT

## Abstract

This paper presents innovations in green electronic and computing technologies. The importance and the status of the main subjects in green electronic and computing technologies are presented in this paper. In the last semicentennial, the planet suffered from rapid changes in climate. The planet is suffering from increasingly wild storms, hurricanes, typhoons, hard droughts, increases in seawater height, floods, seawater acidification, decreases in groundwater reserves, and increases in global temperatures. These climate changes may be irreversible if companies, organizations, governments, and individuals do not act daily and rapidly to save the planet. Unfortunately, the continuous growth in the number of computing devices, cellular devices, smartphones, and other smart devices over the last fifty years has resulted in a rapid increase in climate change. It is severely crucial to design energy-efficient “green” technologies and devices. Toxic waste from computing and cellular devices is rapidly filling up landfills and increasing air and water pollution. This electronic waste contains hazardous and toxic materials that pollute the environment and affect our health. Green computing and electronic engineering are employed to address this climate disaster. The development of green materials, green energy, waste, and recycling are the major objectives in innovation and research in green computing and electronics technologies. Energy-harvesting technologies can be used to produce and store green energy. Wearable active sensors and metamaterial antennas with circular split ring resonators (CSSRs) containing energy-harvesting units are presented in this paper. The measured bandwidth of the matched sensor is around 65% for VSWR, which is better than 3:1. The sensor gain is 14.1 dB at 2.62 GHz. A wideband 0.4 GHz to 6.4 GHz slot antenna with an RF energy-harvesting unit is presented in this paper. The Skyworks Schottky diode, SMS-7630, was used as the rectifier diode in the harvesting unit. If we transmit 20 dBm of RF power from a transmitting antenna that is located 0.2 m from the harvesting slot antenna at 2.4 GHz, the output voltage at the output port of the harvesting unit will be around 1 V. The power conversion efficiency of the metamaterial antenna dipole with metallic strips is around 75%. Wearable sensors with energy-harvesting units provide efficient, low-cost healthcare services that contribute to a green environment and minimize energy consumption. The measurement process and setups of wearable sensors are presented in this paper.

## 1. Introduction

This paper discusses and highlights innovation in green electronic and computing technologies. Moreover, this paper covers the main subjects of green electronics and computing engineering. It is especially important to create a green environment for cancer patients. Cancer patients undergo radiation and chemotherapy treatments that significantly weaken their immune systems. Conventional communication and electronic devices contain toxic materials that may endanger cancer patients. Green communication and electronic devices may be used to assist and monitor the health of cancer patients. Moreover, green IoT (GioT) devices may assist cancer patients. In the last fifteen years, several research papers and books on green computing and electronic technologies have been published [1,2,3,4,5,6]. In [1], a computational perspective on green computing and blockchain technologies is presented. The book presents how to identify challenges using a practical approach and also describes how to develop strategies for addressing industry challenges. 

Renewable energy is one of the most important topics in green computing and electronic technologies. Advanced green materials are needed and employed in the industry, and research on green computing and electronic technologies is presented in [2]. Examples of materials used for renewable energy are photovoltaic solar cells, which can be used to produce green energy. Efficient renewable energy systems are discussed in [3]. Several topics in the green computing industry and computing engineering are discussed in [4,5]. In [6], an explanation of how to enhance the energy efficiency of a large-scale computing system is described. Green computing describes the responsible use of computers and computing devices, providing a green environment. Green computing may be considered as the development, research, and production of green computers, involving studies to minimize climate change and air pollution whenever computers operate. Computer manufacturers and electronic companies are facing significant demand from environmental organizations to replace hazardous toxic materials with green materials in their electronic systems. There is a continual global environmental movement to use green electronics and green materials in the electronic industry and in the production of computers. A series of initiatives in several countries require computing and electronic devices to use electronic waste in the production process. The use of polluting materials such as lead, fuel, and plastic is not permitted by green legislators and organizations. Computer and electronic engineers are faced with an increasing demand to design and produce computing devices that are green. The main goal is to use efficient green energy, minimize the number of parts in systems, re-use electronic waste, and use green energy and technologies. Several types of green energy technologies, such as energy harvesting, which may be used in green electronic systems are discussed in this paper. Energy-harvesting devices and theory are discussed and presented in [7,8,9,10,11,12]. The antennas used in RF energy-harvesting modules are discussed in [13]. It is crucial that sensors for electromagnetic harvesting devices are efficient and wideband. Compact efficient wideband antennas for electromagnetic energy-harvesting modules are discussed in [14,15,16,17]. Advanced technologies, such as fractal and metamaterials, are employed to design efficient wideband sensors [18,19,20,21,22,23,24,25,26,27]. The fourth industrial revolution [28] challenges computer researchers, developers, and manufacturers to invest in manufacturing green cellular phones and computers by minimizing the usage of toxic materials and enhancing the reuse of computer and smartphone waste. Novel advanced technologies such as IoT, artificial intelligence AI, robotics, and 3D printing are generating new daily life routines in how people buy, exchange, use, and distribute products. These technologies enhance machine and computer automation, considerably reduce power consumption, and help to create a green environment. The Internet of Things (IoT) enables businesses to monitor, manage, and automate their operations more efficiently and with more control [29]. IoT devices may be part of green electronic and computing systems. Moreover, wearable IoT devices may provide a significant contribution to green healthcare monitoring systems in hospitals and medical centers [30]. Fractal and metamaterial wideband efficient sensors and antennas are an important part of wearable healthcare and IoT devices [31,32,33,34,35,36,37]. Passive and active compact wearable sensors and antennas for medical and IoT applications are discussed in [38,39,40,41,42]. Wearable medical devices may monitor and evaluate a patient’s daily health [43,44,45,46]. The sensors and antennas developed and presented in this paper were evaluated using electromagnetic software [47]. There is a good match between the calculated and measured results presented in this paper. Wearable circular polarized antennas for healthcare, 5G, energy harvesting, and IoT systems are presented in [48]. The antennas presented in this paper can be used for various data transmission standards such as Bluetooth, Wi-Fi, 5G, and 6G.

References [49,50] present a survey about the advanced solutions and technologies that can help IoT-enabled smart grids and blockchain devices to be more resilient and secure in overcoming existing cyber and physical attacks. Wearable sensors and antennas can be employed in smart grid and medical applications, see [48,49,50]. In [13,14,51], measurement setups and the measured results of sensors and antennas in the vicinity of the user’s body are discussed and presented. Reviews on RF energy-harvesting modules theory and efficiency are presented in [51,52,53,54,55,56,57,58]. The efficient processing and storage of information is crucial to minimize energy consumption and environmental pollution. The importance of green computing is presented in this paper. Moreover, wearable sensors with energy-harvesting units provide efficient, low-cost healthcare services that contribute to a green environment and minimize energy consumption.

## 2. Green Computing and Electronic Technologies Main Topic

Smart devices and computers have become a major part of our daily life. Everyone owns and use more than one smart computing device. People own trillions of electronic products that consume around 26,000 Terra Watt/hour in 2023. It is crucial to develop green computing devices. By developing and manufacturing energy-efficient computing devices, smart phones, medical electronic devices, servers, and laptops, we can minimize air and water pollution. One of the main goals of computer and electrical engineers and researchers should be to develop and manufacture green computing devices, smart phones, medical electronic devices, servers, laptops, and tablets. Moreover, companies and organizations should dispose of and recycle material waste, unwanted computing, medical, and electronic devices. Every person should contribute to green computing by the responsible and smart use of cellular phones, laptops, computing devices, smart watches, and tablets.

Personal Contribution to Green Electronic and Computing

Green computing is known as the study of designing, developing, engineering, research, producing, using, and disposing of computers and using networks to minimize energy consumption, climate hazards, and air pollution. Computer developers, researchers, designers, and computer companies are investing in the research and design of green computing networks by using green renewable energy, reducing the use of toxic materials, and applying an improved recycling process of computing devices.

Personal Contribution to Green Computing and Electronics Systems

- Buy and use energy-efficient computing and electronic systems, smart devices, and laptops with a green tag and with high energy efficiency.
- Turn off electronics, computers, smart device, and laptops when they are not in use.
- Computing devices consume electricity in standby state. It is advised to turn down the brightness of the computing networks when they are on standby mode.
- Minimize printers’ electricity consumption, as they use a lot of electrical power. Print only when it is necessary and recycle paper. Email and sharing services are green services.
- Use computing networks efficiently. Reduce the runtime and energy required to do computing tasks by using updated software and drivers.
- Recycle computers and electronic devices to generate a green planet.
- In 1992, the Energy Star project initiated the study and the engineering of green computing networks in 1992 by the Environmental Protection Agency, EPA.

Major Activities in Green Electronic and Computing Engineering

- Green energy—Developing renewable green energy devices. Reducing energy consumption of computing networks, laptops, and smart devices. Minimizing energy consumption by employing solar and wind energy.
- Green waste disposal: Electronic waste recycling. Reusage of unwanted computing devices, smart phones, laptops, smart sensors, tablets, and other electronic devices.
- Green development and design: Developing and manufacturing energy-efficient computing networks, smart devices, laptops, servers, and other computing devices.
- Energy harvesting—Employing energy-harvesting systems and technologies to reduce the electrical energy consumption of computing networks and smart devices.
- Green manufacturing of smart devices: Manufacturing green computing networks and using recycled computing modules. Minimizing electronic waste in the manufacturing process of computers, laptops, tablets, smart devices, and other computing devices.

The environmental disaster that our world is facing at this decade can be solved only by employing green electronic technologies. The climate disaster has resulted in severe storms, hot temperatures, ocean and sea pollution, floods, severe air pollution, and water pollution. Green electronic and computing systems, the production of green energy, recycling electronic and computers waste, and the production of green electronic devices are the main topics and challenges in green electronic engineering. The planet suffers from severe environmental changes, the depletion of groundwater reserves, severe droughts, and air and water pollution. There is a continuous growth in the number of countries that suffer from severe droughts, air pollution, water pollution, and seawater pollution. The earth temperature rises, and it causes seawater levels to rise. Researchers predict that several big cities will disappear under the ocean water. Environmental changes are almost irreversible and cause diseases, virus transmission, and the extinction of some types of creature. In the last twenty-five years, the electronic and computing companies, airplane and satellite industry, cars, and communication industry have drastically consumed and ruined the universal finite resources. Climate changes, polluted rivers, polluted air, and water affect children and adult health. Chemical toxics, plastic waste, and hazard materials kill birds, animals and sea, river, and ocean creatures. Sea habitats and fishes swallow toxic and hazard materials. Unfortunately, toxic materials and nylon particles make their way to our plate and into our body.

## 3. Green Environment and Electronics Technologies

The computer and communication industries are responsible for 2% to 6% of the overall global greenhouse gas emissions. Green organizations and Western governments apply continuous pressure on computer and electronics companies to use green electronic and computing technologies. Green technologies have seen rapid growth in the last twenty years. Moreover, smart electronic devices, communication products, and computing companies are under continuous pressure from green organizations and legislative authorities to produce green devices and to use green materials. Green organizations apply continuous pressure to consume green products, green materials, and green energy and to produce green computing devices. The use of lead, iron products, plastic products, copper, and other hazardous materials should be minimized or not allowed to reduce air and water pollution. The carbon emissions and energy consumption of the global electronic industry should be minimized to decrease environmental disasters and to prevent a catastrophic irreversible planet disaster. Green computing and electronics seek to minimize the carbon emissions price tag. Computer engineers and developers should develop efficient electronic devices to reduce energy consumption and the quantity of heat these devices emit to reduce the carbon emissions of computers and laptops. Moreover, computing and electronic system developers should improve the lifetime of computers and laptops to reduce electronic waste.

Major Activities, Objectives, Challenges, and Targets in Green Technologies

- Developing and using green energy.
- Using green materials and avoiding toxic and hazardous materials.
- Minimizing polluting energy usage as the main objective of green electronics.
- Reducing carbon emissions.
- Developing efficient protocols to improve computers’ performance.
- Management and recycling of electronic and computer waste.

Generating a Green Electronic and Computer Industry

Energy efficiency and green engineering in computing centers and in other IT facilities may be improved by employing the following recommendations.

- Install green computers and computing systems that are energy efficient.
- Reduce the heat emitted by computing devices.
- Use and buy energy-efficient computers, desktop systems, smart phones, servers, laptops, and other computing devices.
- Reduce heat and energy consumption in offices and computer companies’ buildings.
- Use electronic and communication devices with energy-harvesting units.

## 4. Cloud Storage and Green Computing

Cloud computing refers to a service that provides computing services and storage over the internet instead of via hardware like a USB drive, hard disk, or other storage device. These services supply shared computing networks, computers, data storage devices, and other computing facilities. Cloud computing become essential to companies that want advanced alternative ways to store, manage, and process complex data without being tied to a local computing network. Cloud computing providers supply services that provide access to shared computers, servers, data storage, and other services. These services may be easily controlled, accessed, inspected, and compiled with minimum labor effort and cost. These services provide companies and users the options to run and manage their computer programs, store programs and data, and simulate company programs in a private cloud computing facility. These computing services allow institutions and organizations to access efficient and fast computers and reduce computing costs such as those of servers, computers, and expensive computer programs. Cloud computing providers may use advanced computing resources to run complex computer programs and to execute a trillion computations per millisecond. These computing services cut users’ and institutions’ energy and computing costs and provide companies with green computing services. These computing services allow institutions and users to concentrate on their main tasks and minimize the cost of computing staff and computing networks. The main types of cloud storage are private cloud storage, public cloud storage, and hybrid cloud storage.

Cloud Computing Activities and Services

- Webex, Zoom, and Teams are cloud software networks for audio and video meetings.
- Salesforce is an SaaS services provider: Software-as-a-Service, SaaS.
- IaaS, online virtualized computing services and infrastructure, provided by IBM and others.
- Paas, Platform-as-a-Service.
- Dropbox and OneNote provide file sharing and data storage.
- Civis Analytics and several other companies provides big data analysis.
- Cybersecurity services and software are provided by several companies.

Netflix is using cloud storage services. Movies and TV programs are stored in the cloud. The company can produce more TV services online and has more customers. The company may automatically decrease or add online storage amounts based on customer’s demand.

## 5. Green Wearables, Electronics, Computing Devices, and Technologies

In 2023, wearable devices which help users to collect, monitor, and analyze real-time personal information became extremely popular. People wear wearable devices to monitor, evaluate, and transmit their data. It is crucial to develop green wearable devices to reduce environmental hazards. Wearable device designers and developers should develop efficient wearable sensors to reduce energy consumption and the quantity of heat these devices emit to reduce the carbon emissions of wearable devices. Moreover, the wearable devices presented in this paper contain an energy-harvesting unit to recharge the device battery and to produce green energy. Wearable devices designers and developers should use green materials and improve the lifetime of wearables to reduce electronic waste. Wearable devices can monitor medical data from the heartbeat rate, temperature, sleep behavior, and sweat. Wearable devices can save users’ lives, especially the elderly and children. The information collected by wearable sensors can be transferred to computing medical centers or stored in the cloud.

Benefits of Wearable Devices and Technology

Wearable devices are exposed to sunlight and to electromagnetic energy that propagates in free space. Energy-harvesting modules can be assembled in wearable devices to produce and store green energy.

- By using wearable medical devices, physicians may rapidly evaluate and diagnose patient health.
- Athletes may improve their sports records by using wearable sports sensors. The collected health data assist the athletic and sports companies to improve fitness and training processes.
- The health and productivity of working staff may be improved by using wearable sensors.
- The communication between company personnel and workers may be improved by using wearable sensors that may monitor staff activities. Wearable devices improve companies’ safety procedures and can improve the daily life quality of the company workers. Happier and healthier workers increase the productivity rate of employees. This fact saves companies significant work expenses.
- Wearable devices and sensors are significant part of IoT systems and networks.
- Wearable sensors and antennas can monitor medical centers and hospitals’ daily procedures such as patient blood pressure, patient heartbeat, and patient temperature.

Green Wearable technologies applications and examples

Medical wearables. Wearable sensors and antennas can monitor medical centers and hospitals’ daily procedures such as patient blood pressure, patient heartbeat, and patient temperature.

Health monitoring. Wearable sensors can monitor and track personal information about the user health parameters such as the body temperature, sweat, heartbeat, blood pressure, and calorie budget. The coronavirus pandemic, during 2019 to 2021, increased the growth of the medical wearable sensor industry. People consumed more smart medical sensors to monitor their health and the spread of the COVID-19 virus and other infections’ spread.

Wearable military devices. Soldiers’ activities may be tracked by using wearable military sensors. Boot inserts can examine how well the soldiers are using military equipment. Wearable sensors can examine and test soldiers’ performance and behaviors in several conditions and environments.

Athletic and sport devices. Athletes may improve their sports records by using wearable sport sensors. The collected health data assist the athletic and sports companies to improve fitness and training process. Athletes may use wearable athletic sensors that are part of the sports products such as shoes, bats, hats, and balls. Bluetooth and GPS devices provide online data to sports personnel to track and analyze athletes’ health and sports records. Wearable sport sensors and products are used to monitor various parameters of the athlete such as their blood pressure, sweat, heartbeat, and other athletic records.

**Figure 1:**
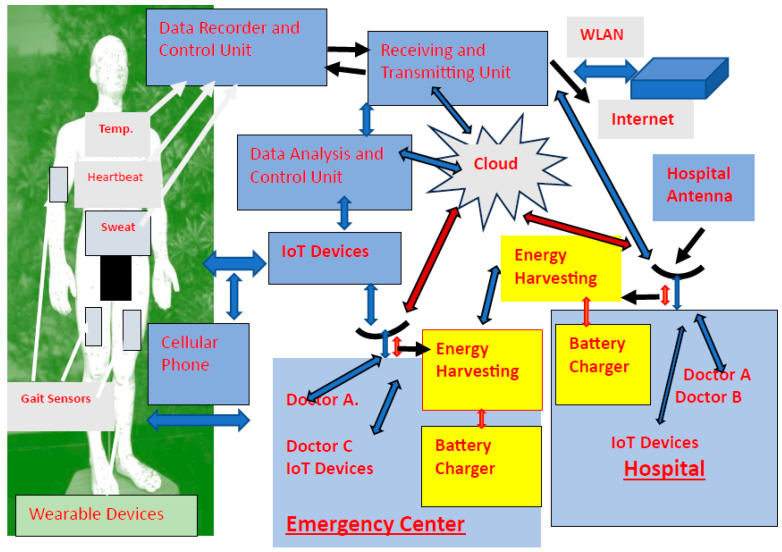
Green medical monitoring system with energy-harvesting modules and IoT devices.

**Figure 2:**
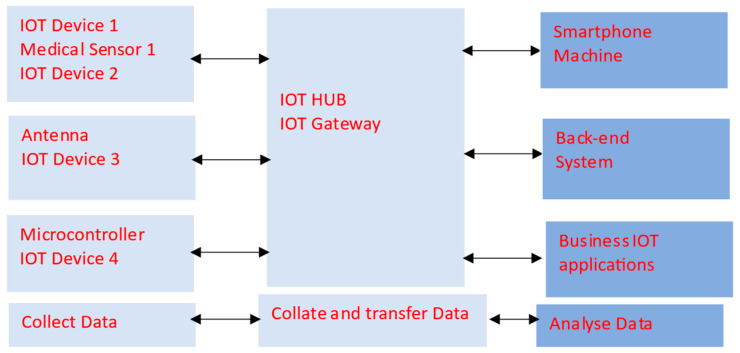
Block diagram of an IoT system with medical devices.

The application of WBANs in healthcare centers, with energy-harvesting units and IoT devices, where the medical parameters of large numbers of patients are constantly being monitored is presented in Figure 1. A block diagram of an IoT system with medical devices is presented in Figure 2.

## 6. Green IoT, GIoT

Green IoT (GIoT) systems and devices use energy-efficient processes and products to obtain a green environment [43,48,49]. The rapid growth of the IoT industry resulted in improving the connectivity between different systems and machines worldwide. GIoT has contributed to minimizing energy consumption by converting conventional systems to green modern devices, giving them the ability to collect information which can be evaluated to provide efficient green electronics and a green environment. Green IoT concentrates on the green development of IoT devices, the green production of IoT devices, green disposal, and the recycling of IoT devices to achieve a green and clean environment. IoT provides smart technology which results in a green environment by using efficient green energy, green materials, and recycling electronic waste. IoT technology reduces air pollution, carbon emissions, and waste. IoT technology has made a significant contribution to the smart homes industry, smart medical devices and systems, smart cities, smart agriculture, and smart electronic systems. IoT improves the quality of life and generates a green environment. The continuous growth in the production of millions of IoT devices has resulted in a huge increase in electrical energy consumption that pollutes the environment. IoT products mostly employ toxic batteries that should be frequently replaced. Battery waste is toxic and pollutes the environment. IoT devices should use green renewable energy sources.

An energy-harvesting unit should be used to recharge IoT devices. Green IoT devices use green efficient energy that reduces energy consumption and minimizes carbon emissions. Cloud green computing networks and green smart sensors have become a significant part of green IoT devices that use green renewable energy. Artificial intelligence technology that uses efficient advanced algorithms provides IoT systems and devices that are user-friendly, smart, and green.

Green IoT Applications

Green energy-efficient homes. IoT networks monitor homes’ device usage by employing electronic monitor modules with online reporting capabilities and remote energy monitoring.

Heat and cooling management. IoT devices monitor heat and cooling appliance usage to reduce energy consumption.

Medical and healthcare monitoring systems. IoT systems can be employed to monitor users’ health. IoMT, the Internet of Medical Things, improves patients’ healthcare, personalized therapies, and safety. Advanced communication systems and devices, such as Bluetooth, RFID tags, and other RF communications technologies, provide a green environment and private wearable healthcare services.

Real-time power usage monitoring and smart utility meters. IoT devices may gather, store, and communicate with wireless communication devices and monitor energy consumption.

Waste management. IoT devices may be used to monitor waste management.

Machine-to-machine, M2M, IoT devices. Smart M2M IoT devices such as refrigerators, washing machines, solar water heating, and utility meters to parking spaces are connected to sensors and can communicate with one another, taking measurements, and they can analyze the collected data.

Green RFID tags, green sensors, green computing centers, and cloud computing services are technologies used by green IoT devices. RFID tags consist of a compact microchip assembled in an adhesive sticker with a transceiver that may receive and transmit data. RFID tags may be used to reduce vehicle pollution emissions and to minimize energy consumption in buildings.

Future Challenges of Green IoT

The major future challenges of green IoT are summarized in the following paragraph.

- Developing energy-saving techniques for IoT devices.
- Developing efficient energy-harvesting modules for IoT devices.
- Developing efficient, compact energy-storage modules such as green batteries, fuel cells, and printed batteries.
- Developing efficient, low-cost green networking and communication for IoT devices.
- Developing green computing networks and cloud services for IoT devices.
- Developing smarter M2M machines that use green renewable energy.
- Developing smart efficient protocols and software for IoT devices.

**Figure 3:**
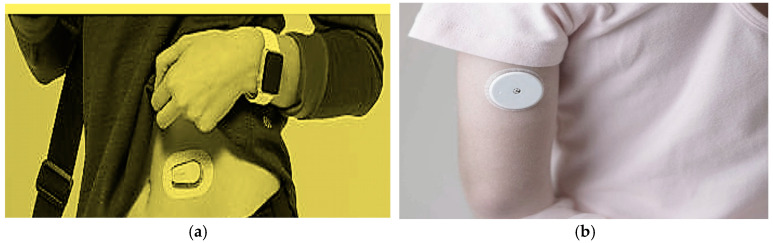
(**a**) Wearable IoT device to monitor glucose rates. (**b**) Wearable device to monitor glucose rates using NFC technology.

A wearable IoT device to monitor sugar rates is presented in Figure 3a. A sensor automatically measures sugar rates and transmits it to a smartphone. Figure 3b presents a wearable sensor that provides remote measurement of blood glucose levels using NFC technology and a mobile phone or NFC reader. The sensor transmits data at 13.56 MHz.

## 7. RF Energy-Harvesting Units and Systems

### 7.1. Introduction to Electromagnetic Energy-Harvesting

RF energy-harvesting devices capture RF fields traveling in the air. RF energy may be harvested by antennas to generate electricity that may charge electrical systems, smart devices, medical sensors, and other wearable sensors. Wideband and ultra-wideband sensors may collect RF energy radiated by several communication systems. Due to low-power densities in the air, efficient wideband antennas are crucial in energy-harvesting systems. The sensors should radiate at a specific frequency band and polarization. Antennas with a wide beam width radiation pattern are required. Many types of harvesting antennas were used, see [4,5,6,7,8,9]. The amount of RF waves in free space increases every day. Everyone men, women, and young kids owns a smartphone, smart device, tablet, or smart watch. The amount of radio waves in the air in 2023 was 100 Exabytes, EB, per month. The expected amount of radio waves in the air in 2028 is expected to be 180 Exabytes per month. Table 1 presents the expected amount of radio waves in free space from 2016 up to 2025. The 5G forecast will account for up to 50% of the global mobile data traffic by 2025. Table 2 presents the net electricity consumption in Terra Watt/hour from 1985 to 2022. Table 3 presents the energy sources used in harvesting devices. The frequency range from 0.1 GHz to 5.6 GHz is used by communication services such as smartphones, television, GSM, IoT, wireless local area networks, WLAN, and Wi-Fi. Wireless communication devices operate at frequencies from 600 MHz to 2.8 GHz. Medical systems operate in frequencies from 150 MHz to 1.2 GHz. WLAN systems operate at frequencies from 5.4 GHz to 6 GHz. The huge increase in the use of computing devices is presented in Table 4. The worldwide data center capacity from 2016 to 2021 in Exabytes is listed in Table 4. The worldwide data center capacity doubled from 850 Exabytes to 1750 Exabytes. The data presented in Table 4 are related to the huge increase in electricity consumption presented in Table 2. Net electricity consumption has increased by 5500 Terra Watt/hour in the last decade. This information explains the significance of renewable energy to protect the environment.

### 7.2. Electromagnetic Energy-Harvesting Programable Arrays and Systems

**Figure 4:**
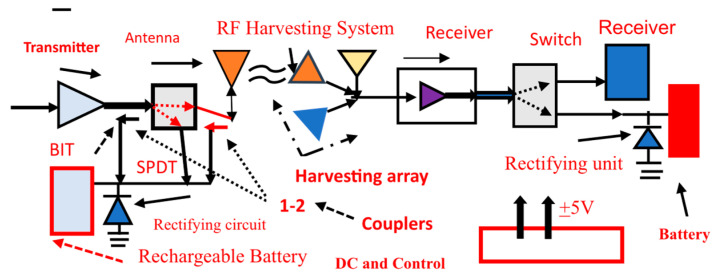
Dual-mode programable array energy-harvesting system 0.1 to 18 GHz.

**Figure 5:**
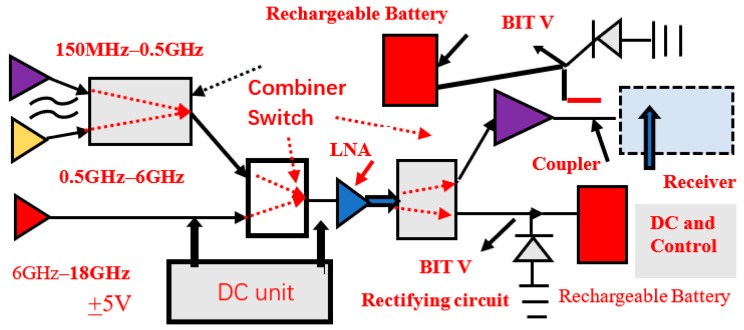
Programmable communication receiver with energy-harvesting units, 0.15 GHz to 18 GHz.

Figure 4 presents the concept of electromagnetic energy harvesting. Electromagnetic far fields are inversely proportional to the distance from the radiating element. Electromagnetic far fields decay as the distance from the radiating element is increased. The radiated RF power from a commercial communication system is around 20 dBm to 30 dBm, so the harvested power from commercial RF systems can be lower than 0.2 mW/cm^2^. An array with three antennas may harvest RF power from UHF frequencies up to Ku frequencies, 0.05 GHz to 18 GHz. The captured electromagnetic power, from each antenna, may be summed and converted to DC energy. The RF power amount in public areas, medical centers, markets, malls, and hospitals may range from 1 µW/cm^2^ to 5 mW/cm^2^. For example, if the RF radiating antennas are close to the harvesting module, the electromagnetic energy captured by the harvesting unit will be higher. Table 5 presents the measured harvester efficiency as a function of the input collected power. The harvesting network efficiency decrease for lower values of harvested power is presented in Table 5. In Figure 5, an amplifier amplifies the input power captured by the energy-harvesting unit to improve the efficiency of the harvesting module. The measured results of commercial RF energy-harvesting systems support the results presented in Table 5, see [1,2,3,4,5,6,7]. The major parts of an energy-harvesting system are the radiating elements, antenna feed circuit, rectifying module, and rechargeable battery. Dual-mode electromagnetic harvesting systems are presented in Figure 4 and Figure 5 and the LNA, Low Noise Amplifiers, shown in these figures are part of the RF system. The amplifiers’ DC bias voltages are supplied by the RF system DC module. The RF power transmitted to the built-in test port is around −20 dB and can be converted to the DC power used to recharge electronic devices, medical sensors, and batteries. Smartphones nay transmit around 1 Watt, applying the standard 802.11. The rate of the harvested energy may be computed by employing the Friis transmission formula for a lossy medium and unmatched antennas, and the received energy harvesting may be computed by using Equation (1).

(1) $$P_{r}=P_{t}G_{r}G_{t}(\frac{\lambda }{4\pi r}{)}^{2}\left(1-{\left|\Gamma_{t}\right|}^{2}\right)\left(1-{\left|\Gamma_{r}\right|}^{2}\right){\left|a_{t}a_{r}^{*}\right|}^{2}e^{-\alpha r}$$

The receiving antenna matching losses are depicted as *L_ra_*, and $\Gamma_{r}$ is the reflection coefficient. *L_ra_* = $\left(1-{\left|\Gamma_{r}\right|}^{2}\right)$.

The transmitting antenna matching losses are represented by Lta = $\left(1-{\left|\Gamma_{t}\right|}^{2}\right)$. The attenuation constant is α. The free space loss (*L_p_*) represents the propagation loss in free space. The losses due to attenuation in the atmosphere are given by *L_a_* = $e^{-\alpha r}$. The polarization losses are *L_pol_* = ${\left|a_{t}a_{r}^{*}\right|}^{2}$. The total loss is Ltotal.

$${Ltotal=L}_{p}L_{a}L_{ta}L_{ra}L_{pol}L_{o}L_{r}$$

where $L_{p}={\left(\frac{4\pi R}{\lambda }\right)}^{2}$. The received power is computed as $P_{r}=\frac{P_{t}G_{t}G_{r}}{L_{p}}$.

(2) $$P_{r}=\frac{P_{t}G_{t}G_{r}}{Ltotal}$$

$\Gamma_{t}$ is the reflection coefficient of the transmitting antenna. *P_t_* = Pout/*L_t_*.

*P_t_* is the transmitting antenna power. *L_t_* is the loss between power source and the antenna.

*EIRP* is the effective isotropic radiated power, where *EIRP* = *P_t_G_t_*. *G* is the gain in dB. *L* is the loss in dB.

(3) $$P_{r}=\frac{P_{t}G_{t}G_{r}}{Ltotal}=\frac{EIRP\times G_{r}}{Ltotal}=\frac{P_{out}G_{t}G_{r}}{Ltotal}$$

which may be written as $G=10\cdot \log\left(\frac{P_{out}}{P_{in}}\right)\;\mathrm{dB}$, $L=10\log\left(\frac{P_{in}}{P_{out}}\right)\;\mathrm{dB}$.

*P_r_* in dBm, the harvested power, is calculated as shown in Equation (4). The electromagnetic energy transmitted by computers’ memory cards may be up to 100 mW. The electromagnetic energy transmitted by smart devices may be up to 1 W.

**Figure 6:**
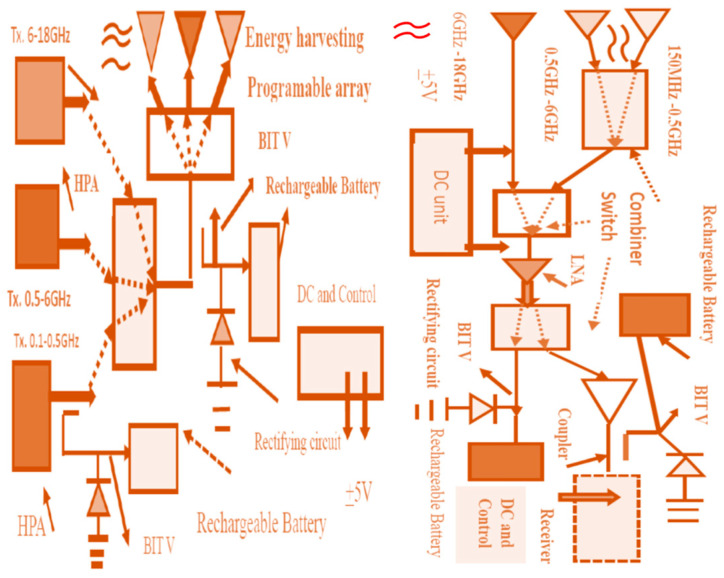
Transceiver with an energy-harvesting programable array system, 0.15 GHz to 18 GHz.

Figure 5 illustrates an ultra-wideband receiving array, 150 MHz to 18 GHz, with an energy-harvesting module. A wideband transceiver is shown in Figure 6, 150 MHz to 18 GHz, with energy-harvesting modules. Figure 5 and Figure 6 presents a wideband transceiver with three wideband antennas. The first antenna operates from 0.15 GHz to 0.5 GHz. The second antenna operates from 0.5 to 6 GHz. The last antenna operates from 6 to 18 GHz. The transceiver systems can operate from 150 MHz to 18 GHz. In several communication systems, part of the RF power, −10 dB to −20 dB, is coupled to a test port. This RF energy may be converted to DC power and can be employed to produce electricity.

(4) $$P_{r}=EIRP-L_{total}+G_{r}$$

**Figure 7:**
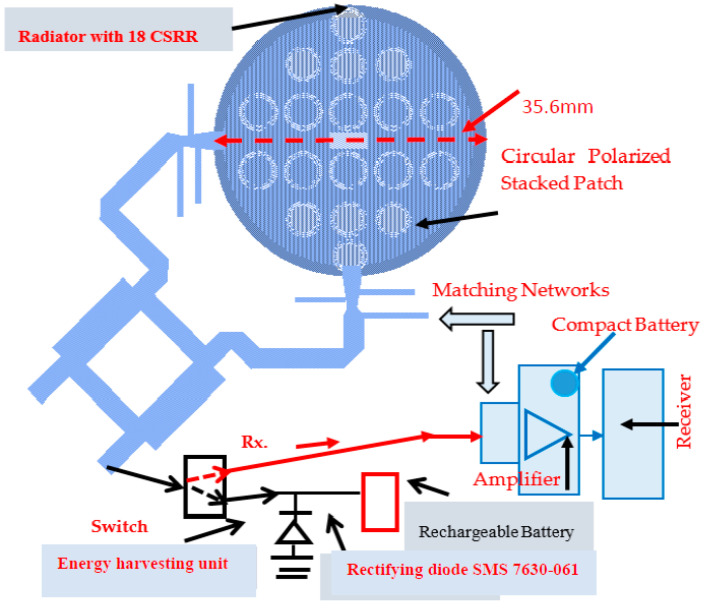
Metamaterial active receiving antenna, circular polarized, with an energy-harvesting module.

**Figure 8:**
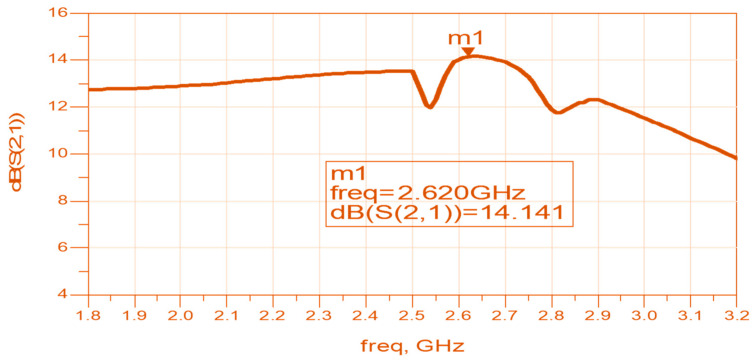
The metamaterial active sensor gain, S21.

**Figure 9:**
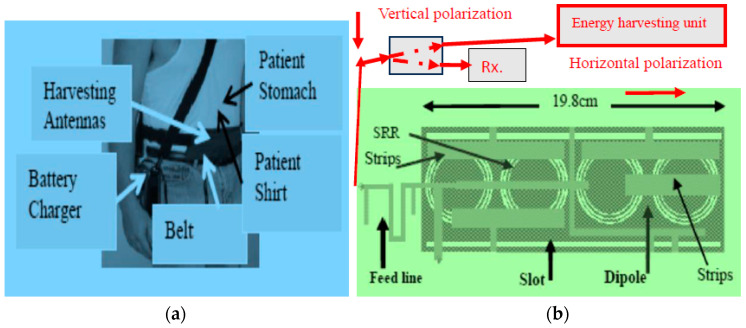
(**a**) Wearable medical sensors with an energy-harvesting system for IoT, 5G, and healthcare applications. (**b**) Wideband dual polarized wearable metamaterial antenna with an energy-harvesting unit.

**Figure 10:**
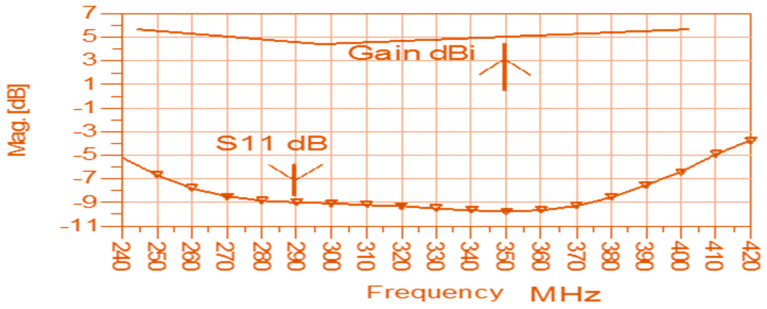
Gain and S11 of the dual polarized antenna with metallic strips and a CSRR.

A control and power supply module supplies the amplifier’s bias voltages and operates the control commands to the switching matrix shown in Figure 5 and Figure 6. As shown in Equation (1), the polarization of the receiver should match the polarization of the transmitter. In some RF systems, the polarization may vary and is not always defined. It is recommended to use circular polarized antennas or dual polarized antennas in these systems. Moreover, to maximize the amount of harvested energy it is important that the receiving antenna will be circular polarized or dual polarized. Figure 7 presents an example of a circular polarized metamaterial sensor with 18 CSSRs, with an RF harvester. The RF harvester and the antenna are self-powered sensors. The circular-polarized stacked double-layer circular patch is etched on a 1.6 mm-thick, Duroid 5880, substrate with a dielectric constant of 2.25. The circular patch radius is 17.8 mm. The antennas’ dimensions were optimized by employing RF design software [47]. An input-matching network matches the antenna to the LNA, Mini-Circuit amplifier TAV541, see Figure 7. The LNA output port is matched to the system. The LNA electrical characteristics are given in the company datasheet. The bias voltages to the active antenna are supplied by the system DC unit. The matching networks are printed on the antenna feed network board. The matching network’s dimensions are 25 × 25 mm. The measured bandwidth of the matched sensor is around 65% for VSWR, better than 3:1. The sensor gain is 14.1 dB at 2.62 GHz and decreases to 10 dB at 3.2 GHz, as shown in Figure 8. The active antenna noise figure is around 1.3 dB for frequencies from 1 GHz to 3 GHz. Wearable medical devices with a harvester for 5G, GIoT devices, healthcare, and 6G devices may be assembled on the user body as presented in Figure 9a [13]. A wideband wearable metamaterial antenna with four CSRRs is presented in Figure 9b. The sensor dimensions are 19.8 × 4.5 × 0.16 cm. The antenna operates from 0.15 GHz to 0.55 GHz with a 5.7 dBi gain and 90% efficiency, see Figure 10.

## 8. Electromagnetic Energy-Harvesting Modules

Figure 4 presents an energy-harvesting module with an antenna, rectifying circuit, and rechargeable battery. The harvester and the antenna are self-powered modules. The electromagnetic energy, AC, is converted to DC power by using a rectifier diode.

**Figure 11:**
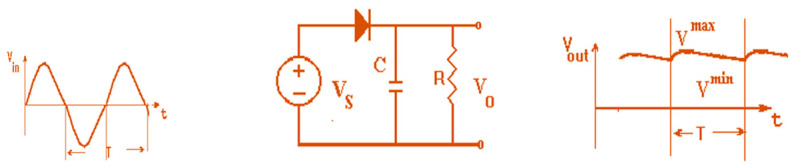
Diode voltage half-wave rectifier with a capacitor, half-wave.

**Figure 12:**
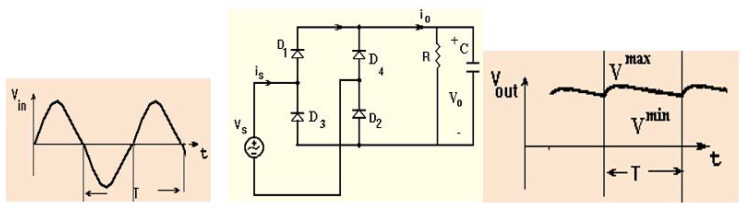
Full-wave diode bridge rectifier, with a capacitor.

Harvesting units usually use a half-wave rectifier or a full-wave rectifier [43,44,45,46,59]. Figure 11 describes a half-wave rectifier. The half cycle of the positive electromagnetic waves is converted by the half-wave rectifier. The efficiency of the half-wave rectifier is 41.2%. A full-wave bridge rectifier that converts RF energy to DC energy is shown in Figure 12. The bridge rectifier consists of four diodes D1 through D4. The rectifier output DC voltage $V_{ODC}=2V_{m}/\pi$. By connecting a capacitor in shunt to the resistor, as shown in Figure 11 and Figure 12, the rectifier’s output voltage is improved. The full-wave rectifier efficiency is 81%. The electromagnetic power amount in public centers, stadiums, hospitals, and malls may range from 0.1 µW/cm^2^ to 10 mW/cm^2^. The harvesting system efficiency increases as a function of the RF power collected by the harvesting system as listed in Table 5. The efficiency of the harvesting system may reach 65% for 10 dBm input power.

In active sensors and antennas with energy-harvesting modules, the amplifier amplifies the power captured by the harvester and increases the efficiency of the harvester. The data presented in Table 5 are presented by the harvester’s manufacturer [2,3,4,5,6,7,44,45,46,59]. The electromagnetic energy captured by the harvesting module decrease as the distance from the transmitting sensor to the receiving sensor becomes higher. $V_{ODC}$ is the rectifier output voltage and is written in Equation (5). The rectifier output voltage can be flattened and improved by adding a capacitor in shunt to the resistor and is written in Equation (6). The capacitor works as a low pass filter to flatten the output DC voltage.

(5) $$V_{O,DC}=\frac{1}{2\pi }\int_{0}^{2\pi }V_{O}^{MAX}\sin(\omega t)d\left(\omega t\right)\begin{array}{cc} ; & \omega =2\pi f \end{array}\;V_{O}=V_{S}-V_{DON}\approx V_{S}\begin{array}{cc} ; & {V_{O}}^{MAX}=V_{m} \end{array}\;V_{ODC}=V_{m}/\pi$$

(6) Vripple=Vr=Vmax−Vmin=VDCfCR

where τ=RC≪T. The half-wave rectifier efficiency is 40.6% as given in Equation (7). The diode resistance *rf* is extremely low compared to the resistance *R*.

(7) $$\eta =\frac{D\;Coutput\;power}{RF\;power}=\frac{{\left(\frac{I_{m}}{\pi }\right)}^{2}R}{{\left(\frac{I_{m}}{2}\right)}^{2}(R+rf)}\sim 0.406$$

The bridge rectifying circuit, usually used for DC power supplies, employs four diodes, D1 through D4, as presented in Figure 12. The rectifier output DC voltage $V_{ODC}=2V_{m}/\pi$. The rectifying circuit output voltage can be improved and flattened by using a shunt capacitor in parallel to the resistor.

The usage of power cords and the need to replace batteries daily may be eliminated by using an RF harvester. Equation (8) verifies that the full-wave rectifier efficiency is 81.2% and only 81.2% of the input AC power is converted into DC power.

(8) $$\eta =\frac{Converted\;DC\;power}{RF\;power}=\frac{{\left(\frac{{2I}_{m}}{\pi }\right)}^{2}R}{{\left(\frac{I_{m}}{2}\right)}^{2}(R+rf)}\sim 0.812$$

The rectifying diode may be a semiconductor Schottky diode, which have a low forward voltage drop and a very fast switching time. The voltage drop of the diode is around 0.15 to 0.5 V which improves the system efficiency, and the switching speed is higher. For example, the Skyworks Schottky diode series SMS-7630 can be used due to their extremely low capacitance, low forward voltage, low series resistance, and fast switching response. The Schottky diode SMS-7630-061 was employed in this research as the rectifying diode. The power conversion efficiency, PCE, achieved with the metamaterial antenna is presented in Figure 9b, and the full-wave harvesting unit is around 75%. By using a voltage doubler, the output voltage of the rectifying circuit may be doubled and the efficiency of the energy-harvesting module may be improved.

Voltage Doublers

**Figure 13:**
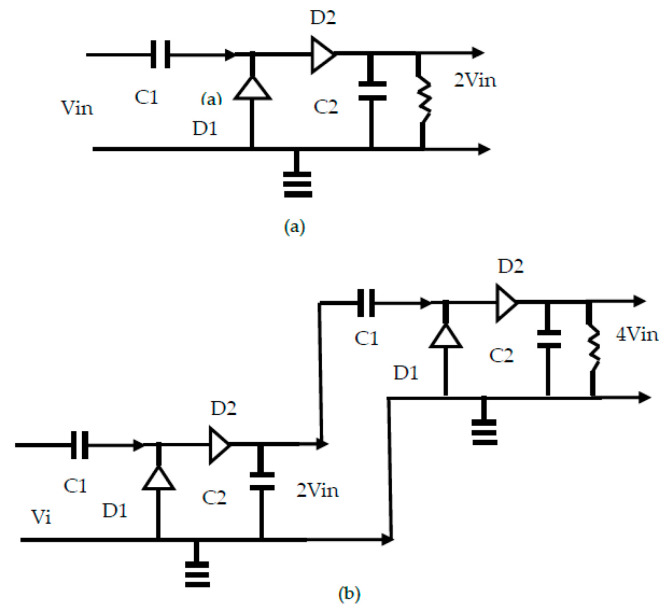
(**a**) Single-stage voltage doubler. (**b**) Multistage voltage multiplier.

Voltage doublers and Dickson multipliers are used to increase a low voltage battery supply to the voltage needed by the integrated circuit. The Dickson multiplier is shown in Figure 13. The single-stage voltage multiplier function as a full-wave rectifier and converts the input AC voltage to a DC voltage. However, compared to the half-wave rectifier, the average DC voltage is doubled. Figure 13a presents a single-stage voltage doubler. The output voltage is 2 Vin. Figure 13b presents a multistage voltage doubler. The output voltage is 4 Vin. During the positive input half cycle, the capacitor C_1_ is charged through diode D_1_. During the negative input half cycle, the voltage of C_1_ adds with that of the source, thus charging C_2_ to 2 Vin through diode D_2_ and discharging C_1_ in the process. In the multistage doubler, the input voltage to the second stage is 2 Vin, so the output voltage will be 4 Vin. The disadvantage of the multistage rectifiers is the decrease in the efficiency which degrades for every additional stage, since the efficiency of every single stage is a multiplying factor smaller than one.

**Figure 14:**
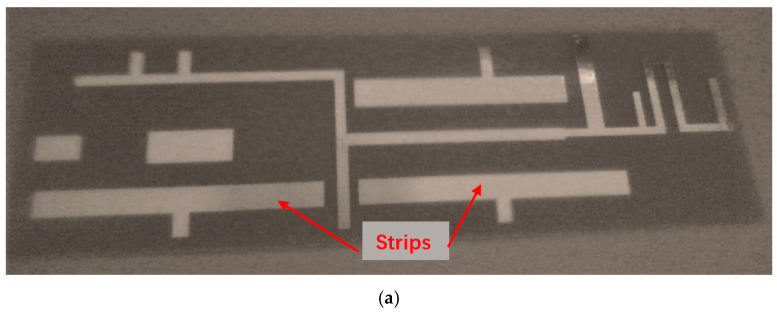

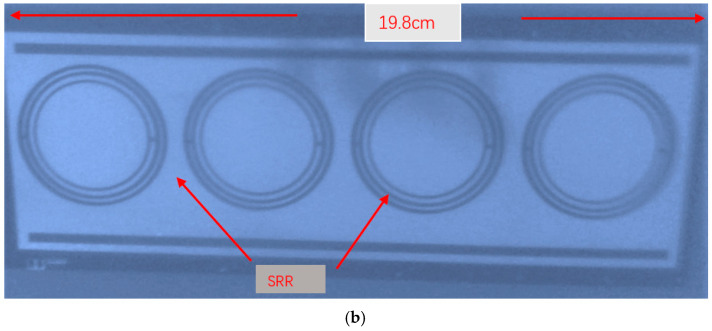
Fabricated efficient wideband metamaterial sensor with a harvesting module. (**a**) The antenna matching network with metallic strips. (**b**) Dual polarized metamaterial sensor with a harvesting module.

The available input power to RF energy-harvesting systems is extremely low. The overall RF-to-DC conversion efficiency depends directly on the selected rectifying circuit. Where the available captured AC power is high, the use of multistage rectifiers results in an increased DC voltage level, although the overall power efficiency is decreased. The evaluation of efficient miniature low-cost RF energy circuits is crucial in the industry of RFID devices and badges. Passive RFID devices may be converted to active RFID devices and tags. Active RFID devices and tags have an improved lifetime and increase the read range. In [50], an energy-harvester is reported that consists of a metamaterial antenna with SRR1 coupled to SRR2. The rectifying circuit is a voltage doubler. At a 2 cm distance, the maximum harvested DC voltage is around 0.5 V at an input harvested RF power of 8 mW at 2.45 GHz. In [53], for an RF energy-harvester at a 20 cm distance, the maximum output converted DC voltage may be around 0.98 V at an input RF power of 0.1 W at 2.45 GHz. A flexible meta-surface coupled efficient wireless power transfer system for implantable medical applications is presented in [54]. In this paper, the Schottky diode SMS-7630 is used as the rectifier diode. The authors declare a value of 68% power conversion efficiency (PCE) at 434 MHz. Photos of the fabricated wideband metamaterial energy-harvesting antenna are presented in Figure 14a, b. In [55], a cross-dipole rectenna for wireless energy harvesting at frequencies from 1.8 to 2.5 GHz is presented. The harvesting unit employs a voltage doubler. The Schottky diode SMS7630 is used in this work. The measured power sensitivity of this design is down to −35 dBm and the conversion efficiency is around 55% for a harvested input power of 0.1 mW. In [56], an octagonal rectenna for wireless sensor applications with an energy-harvesting module with a dual Schottky diode, HSMS270B, is presented. The best efficiency of this rectenna is for an input power around 0 dBm for frequencies from 2.4 GHz to 4.2 GHz. The conversion power efficiency varies from 60% to 80% for 0 dBm power.

## 9. Ultra-Wideband 0.4 GHz to 6.4 GHz, Green Slot Antenna with Energy-Harvesting Unit

**Figure 15:**
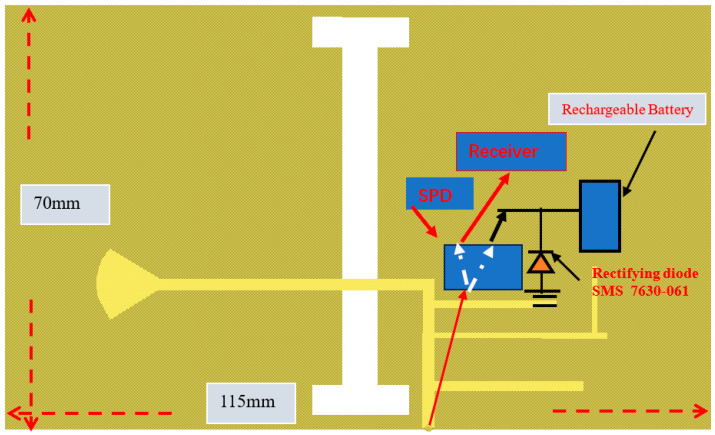
Ultra-wideband green T-shape slot antenna with an RF energy-harvesting unit.

**Figure 16:**
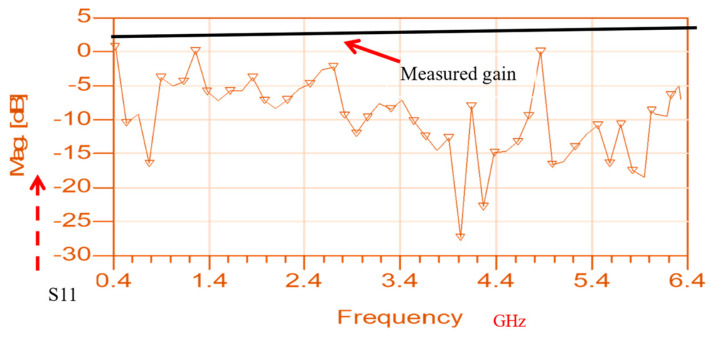
Reflection coefficient of a green ultra-wideband T-shape slot.

**Figure 17:**
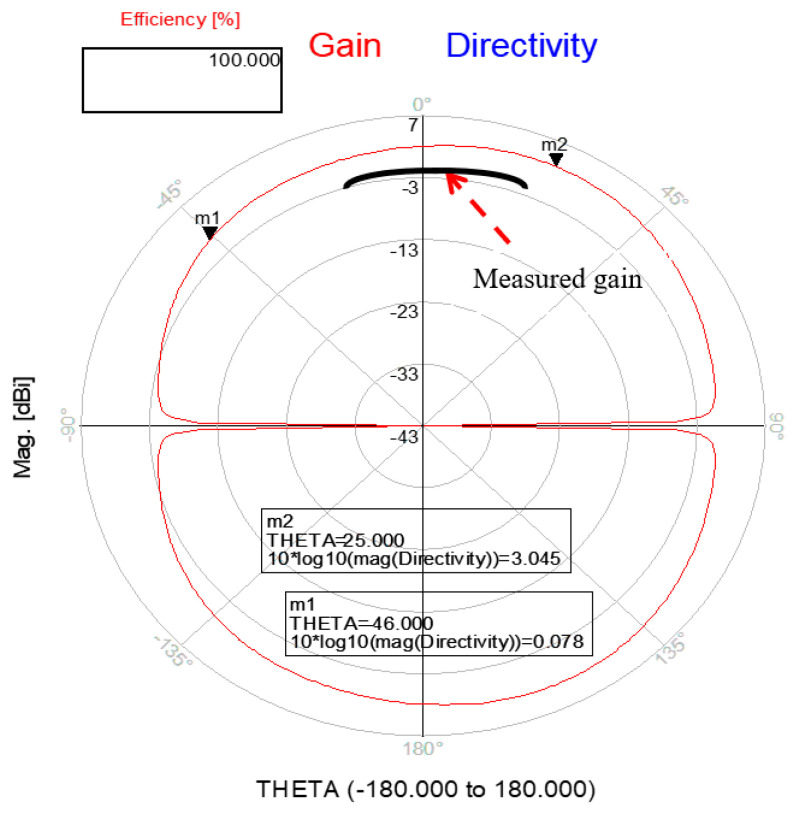
Radiation pattern and measured gain of a green ultra-wideband T-shape slot.

**Figure 18:**
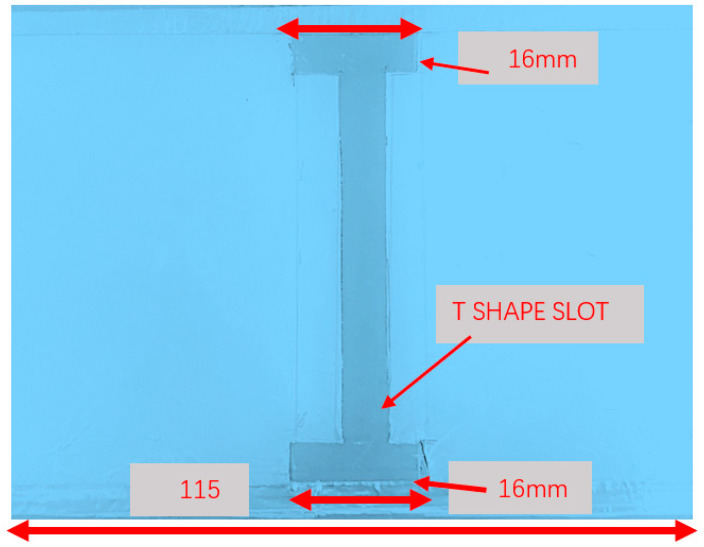
The fabricated wideband energy-harvesting slot antenna, 400 MHz to 6.4 GHz.

An ultra-wideband 0.4 GHz to 6.4 GHz slot antenna with an RF energy-harvesting unit is presented in Figure 15. The slot antenna is etched on a dielectric substrate, Duroid 5880, with a dielectric constant of 2.2 and 1.2 mm thick. The dimensions of the slot antenna are 115 × 70 × 1.2 mm. The antenna electrical parameters were computed by using ADS software [47], version 2021. The reflection coefficient of the wideband T-shape slot antenna is shown in Figure 16. Over around 98% of the frequency range from 0.4 GHz to 6.4 GHz, the antenna VSWR is better than 3:1. The antenna measured gain is 3 dB at 1.5 GHz. The radiation pattern of the wideband T-shape slot antenna at 1.5 GHz is presented in Figure 17. The fabricated ultra-wideband slot antenna is shown in Figure 18. The ultra-wideband, 0.4 GHz to 6.4 GHz, slot antenna can harvest RF energy from cellular phones, IoT devices, and medical devices.

## 10. Green Wideband RF Energy-Harvesting Arrays

**Figure 19:**
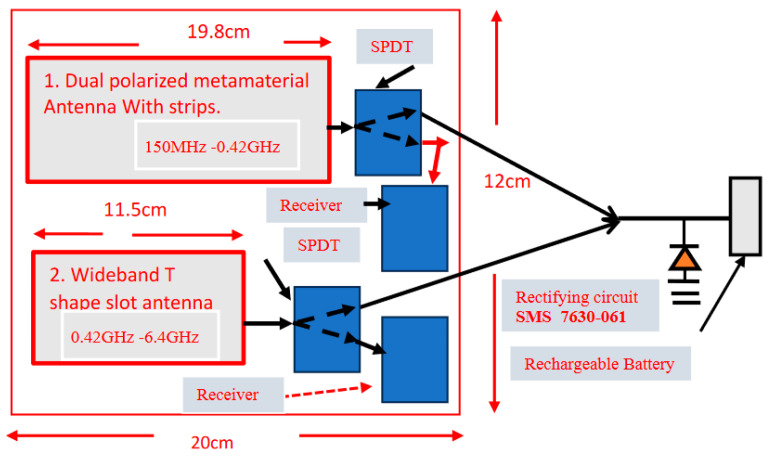
Green wideband RF energy-harvesting panel, 150 MHz to 6.4 GHz.

Most of the radiated electromagnetic energy covers the frequency range from 0.1 GHz to 6.4 GHz. Figure 19 describes a wideband harvesting array with two antennas. The harvesting array size is 200 × 120 × 0.2 mm. The harvesting array may collect energy in frequencies from 150 MHz up to 6.4 GHz as part of healthcare systems, 5G devices, RF, and IoT applications. The wideband harvesting array operates as a dual-mode energy-harvesting module. The antenna is connected to an SPDT that can connect the antenna to the receiver or the harvesting module. The Skyworks Schottky diode SMS-7630 was used as the rectifier diode. If we transmit 20 dBm RF power from a transmitting antenna, with around 6 dBi gain, which is located 0.2 m from the harvesting slot antenna at 2.45 GHz, the output voltage at the output port of the harvesting unit will be around 1 V. The load resistance is 1 kΩ. The progress in the development of wearable antennas’ characteristics from 2010 to 2023, with energy-harvesting modules, is listed in Table 6. The received power by the slot antenna for different transmitted power levels and a distance of 0.2 m and 0.5 m from the transmitter is given in Table 7. The received power by the slot antenna and a comparison between the computed and measured harvested voltages are given in Table 8. The harvesting unit efficiency is around 75%.

A comparison of measured harvester efficiency as function of input power is presented in Table 9. These results were published in the last six years. RF measurements are presented in Section 12.

The simulation and measured results of wearable sensors with energy-harvesting units are given in Table 10. These results were published in the last ten years, and there is good agreement between the simulation and measured results. Measurement setups and the measurement process of wearable antennas with energy-harvesting units have been shown in previous publications [51,52,53,54,55,56,57,58]. Measurements of the wearable antenna by using a phantom are presented in detail in [51]. RF and antenna setups and measurements are presented in detail in [13,14] and also in Section 12.

## 11. Wearable Sensors Measurements and Setups

**Figure 20:**
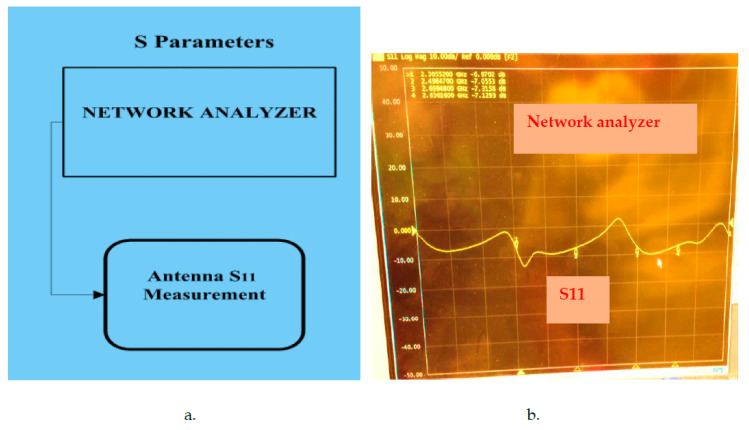
(**a**) Antenna S_11_ parameter measurements block diagram. (**b**) Measured antenna S_11_.

**Figure 21:**
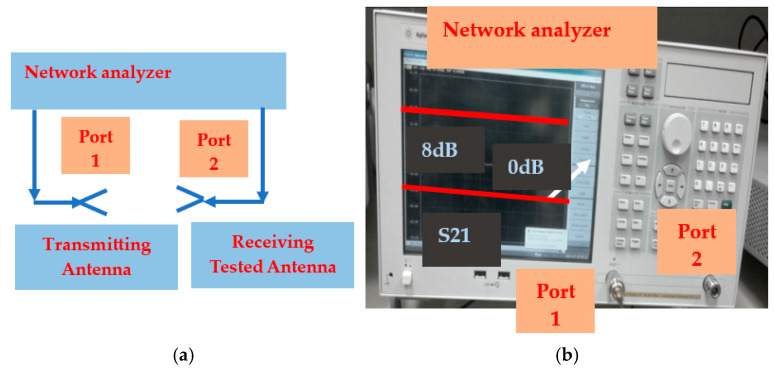
(**a**) Gain measurements. (**b**) Network analyzer S21 measurements.

**Figure 22:**
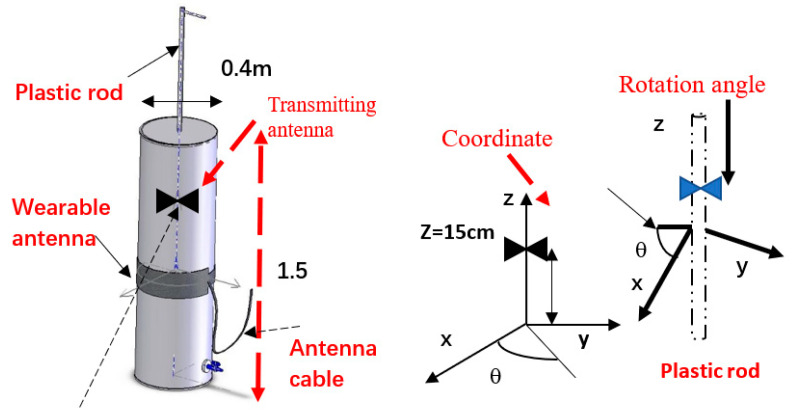
Measurement of wearable sensors using a phantom with a rotated rod.

The wearable antennas and sensors were evaluated on several patients. However, the sensors’ prototypes and measurements during the development process employed a phantom and network analyzer setup. The radiated power meets international standards and does not affect the patient health. The sensor’s reflection coefficient and gain measurements were measured by using a network analyzer and are shown in Figure 20a,b. The network analyzer should be calibrated to two-port calibration as a function of the measured frequency range. S21 measurements measure the gain of the sensor as presented in Figure 21. S21 measurements are two-port measurements. The radiation parameters of wearable sensors are tested and measured by using a lab phantom. The phantom is a dielectric cylinder, usually fiberglass, as presented in Figure 22. The phantom height is 1.5 m. The phantom radius is 0.2 m. The phantom is filled with water, sugar, and salt. Around 90% of the mixture is water. The percentages of sugar and salt in the mixture determine the electrical characteristics of the mixture that may be adjusted to the human body tissues’ electrical characteristics. The phantom may be used to measure electromagnetic radiation from inside or outside the phantom. The phantom contains a plastic rod of 5 mm thickness. The position of the plastic rod inside the phantom may be adjusted. The plastic rod may be rotated. A small transmitting antenna may be attached to the plastic rod at different height positions. The antenna may be rotated in the x-y plane.

**Figure 23:**
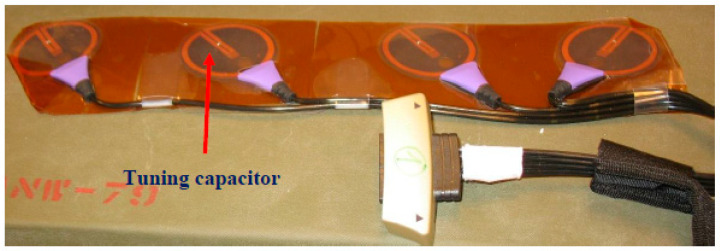
Wearable sensor with four loop antennas rotated +45° and −45°.

**Figure 24:**
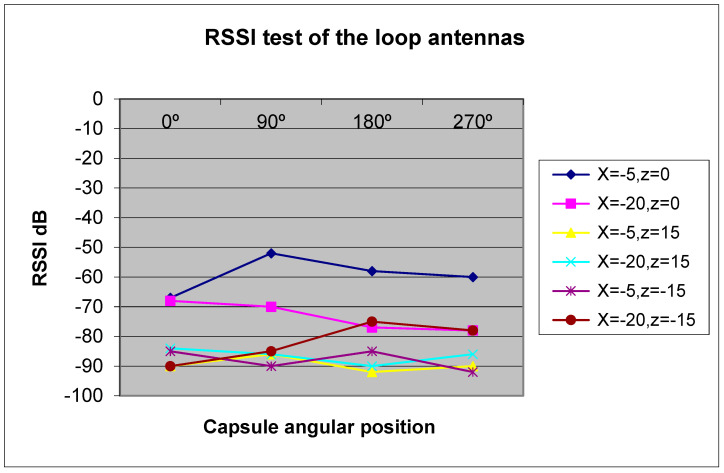
Phantom test results for loop antennas with a sleeve.

A wearable sensor with four loop antennas is shown in Figure 23. The electrical characteristics of the antenna were measured by using the phantom. The wearable antenna electrical characteristics were measured when the height distance, the Z coordinate, between the transmitting antenna and the wearable antenna was 15 cm, 0 cm, and −15 cm. The horizontal position, the X coordinate, of the transmitting antenna was set at −20 cm and −5 cm. The measured results are presented in Figure 24.

**Figure 25:**
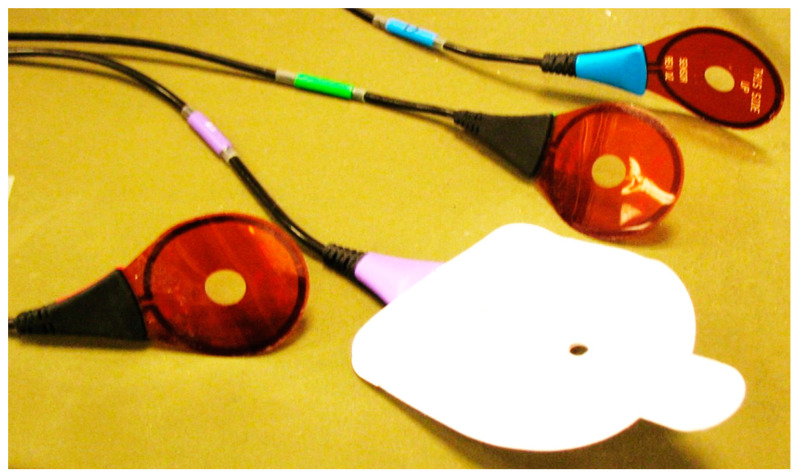
Fabricated loop antennas with and without a sleeve.

**Figure 26:**
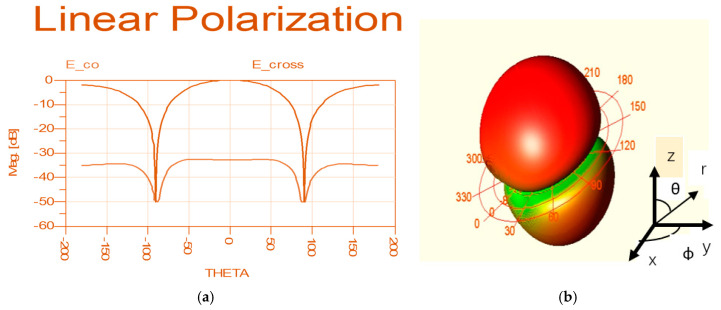
(**a**) Wearable loop sensor on the patient body. (**b**) 3D radiation pattern.

Loop antennas with sleeves are presented in Figure 25. The loop antenna diameter is 50 mm and it is printed on FR4 substrate 10 mm thick. The VSWR of the loop antennas with sleeves is better than 2:1 at 0.42 GHz to 0.46 GHz on the human body. The printed loop antenna radiation pattern at 435 MHz on the human body is shown Figure 26a, and the loop antenna 3D radiation pattern is shown Figure 26b. The loop antenna gain is around 1.5 dBi.

The influence of the human body on the antenna performance is simulated by evaluating the antenna reflection coefficient on the human body. In fat tissues, the dielectric constant is 5; it is 45 in the stomach zone, 41 in the skin tissue, and increases to 128 in the small intestine area [60,61]. The variation in the electrical characteristics of the body tissues affects the electrical performance of the antenna. The sensor resonant frequency is shifted around 2% to 5%, in various locations of the sensor on the patient’s body. If the sugar and salt concentrations in the phantom mixture change, the dielectric constant of the phantom changes. Small changes, less than 5%, will not affect the electrical performance of the wearable antennas. The antennas presented in this paper are wideband antennas. These antennas may be located in several locations on the human body. The simulation and measurements of the wearable antenna by using a phantom are presented in detail in [13,51].

## 12. Conclusions

The world suffers from continuous climate change and disasters such as high temperatures, drought, and flood. The world is suffering from a global rise in Earth’s temperature, increasingly wild hurricanes, typhoons, and floods, air pollution, and pollution of the sea and groundwater. This paper presents innovation in green computing and electronic technologies for 5G and 6G communication services and healthcare devices. It is especially important to create a green environment for cancer patients. The sensors presented in this paper were evaluated and used by several patients. Cancer patients undergo radiation and chemotherapy treatments that significantly weaken their immune systems. Conventional communication and electronic devices contain toxic materials that may endanger cancer patients. Green communication, medical, and electronic devices may assist and monitor cancer patient health. Moreover, green IoT, GioT, and medical devices may assist cancer patients. This research started to detect and assist gastric cancer patients. However, it may be applied to melanoma cancer and other cancer diseases. Unfortunately, the continuous growth in the number of computing devices, cellular phones, smartphones, and other smart devices over the last fifty years has resulted in a rapid increase in climate change. There are several billion devices consuming megawatts of power and many tons of computing and electronic waste. It is severely important to develop efficient “green” devices, technologies, and systems. Green technology engineering is endorsed to minimize air and water pollution. Several types of green wearable low-cost sensors and antennas are presented in this paper. The production cost of these antennas is less than USD 100. Energy-harvesting devices and technologies may be used to generate and store green energy. Wearable active sensors and metamaterial antennas with circular split ring resonators, CSSRs, with efficient energy-harvesting units are presented in this paper. A compact active metamaterial circular polarized sensor with an energy-harvesting unit is also presented in this paper. The measured bandwidth of the matched sensor is around 65% for VSWR, better than 3:1. The sensor gain is 14.1 dB at 2.62 GHz. A wideband dual polarized dipole-slot metamaterial antenna with metallic strips and an energy-harvesting unit was developed in this paper. The antenna frequency range is from 0.1 GHz to 0.55 GHz with a 5.7 dBi gain and 90% efficiency. An ultra-wideband 0.4 GHz to 6.4 GHz slot antenna with an RF energy-harvesting unit is presented in this paper. The antenna measured gain is 3 dB at 1.5 GHz. The Skyworks Schottky diode SMS-7630 was used as the rectifier diode. If we transmit 20 dBm RF power from a transmitting antenna that is located 0.2 m from the harvesting slot antenna at 2.4 GHz, the output voltage at the output port of the harvesting unit will be around 1 V. The Skyworks Schottky diode SMS-7630 was used as the rectifier diode in the harvesting unit of the slot antenna. The power conversion efficiency of the metamaterial antenna dipole with metallic strips was around 75%.

In future research and design, green healthcare monitoring systems should be evaluated to provide green, efficient, and low-cost healthcare services.

## Figures and Tables

**Table 1:** Electromagnetic energy level in the air, EB per month, from 2016 up to 2024.

Year	Level of RF Waves in Air EB per Month	Level of RF Waves in Air EB per Month 5G
2016	5	0
2018	15	0
2020	35	0
2021	60	10
2023	100	25
2024	163	78

**Table 2:** Net electricity consumption per Terra Watt/hour from 1985 to 2022.

Year	Net Electricity Consumption in Terra Watt/Hour
1985	8600
1990	10,400
1995	11,500
2000	13,500
2005	15750
2010	18,750
2012	20,000
2014	21,000
2018	23,500
2020	24,000
2022	25,500

**Table 3:** Energy sources used in harvesting systems.

Energy Source	Type	Efficiency	Harvested Power
Light	Outdoor/Indoor	10~25%	100 mW/cm^2^
Thermal	Human Industrial	~0.1% ~3%	60 µW/cm^2^ ~1–10 mW/cm^2^
Vibration	~Hz—human ~kHz—machines	20~50%	~4 µW/cm^3^ ~800 µW/cm^3^
Electromagnetic	900–2700 MHz Wi-Fi, WLAN	~50%	0.1 µW/cm^2^ 0.001 µW/cm^2^
RF GSM 1 GHz	Wireless GSM	~50%	0.1 up to 1 mW/cm^2^

**Table 4:** Worldwide data center capacity from 2016 to 2021 in Exabytes.

Year	Data Center Capacity in Exabytes
2016	850
2017	1100
2018	1450
2019	1750
2020	2050
2021	2400

**Table 5:** Measured harvester efficiency as a function of the input collected power.

Input Power dBm	Efficiency %	Remarks
−4–−6	8–12	Low Efficiency
−3–−2	28–32	Low Efficiency
−1–+1	48–52	Good Efficiency
2–3	52–56	Good Efficiency
4–5	52–56	Good Efficiency
6–8	56–58	Good Efficiency
9–11	60–65	Best Efficiency

**Table 6:** The progress in development of wearable antennas, with an energy-harvesting module, 2010–2023.

Antenna Type	Gain dB	2010–2015 [7,8,9,10,11,12,13,14,51,52,53,54,55,56,57,58]		Gain dB	2015–2023 [13,51,52,53,54,55,56,57,58]	
		BW%	Effic. %		BW%	Effic. %
Printed dipoles	3	5	70	4	5	80
Patches	3.5	5	70	4	5	80
Stacked patches	7	12	85	7.5	15	85
Metamaterial antenna	3.5–4.5	8–11	45–55	7.5–8.6	40–48	88–93
Metamaterial antenna with strips	-	-	-	5.5–8.5	45–55	88–93
Fractal antennas	4–4.8	3–6	65–75	4–4.8	8–11	80–85
T-shape slot antennas	2.5	10	80	3	0.4–6.4 GHz	85
Circular polarized metamaterial patch	-	-	-	14	1.8–3 GHz	Active Antenna

**Table 7:** Received power by the slot antenna as a function of the transmitted power.

*F *= 2.45 GHz *R* = 1 kΩ	*R *= 0.2 m, *G_r_* = 3 dB *G_t_* = 6 dB, *L_r,t_* = 0 dB	*R *= 0.2 m, *G_r_* = 3 dB *G_t_* = 7 dB, *L_r,t_* = 0 dB	*R *= 0.5 m, *G_r_* = 3 dB, *G_t_* = 6 dB, *L_r,t_* = 0 dB	*R *= 0.2 m, *G_r_* = 3 dB *G_t_* = 5.2 dB, *L_r,t_* = 0.9 dB
*P_t_* (W)	*P_r_* (milli-Watt)	*P_r_* (milli-Watt)	*P_r_* (milli-Watt)	*P_r_* (milli-Watt)
0.1	1.9	2.4	0.302	1.27
0.05	0.94	1.2	0.151	0.635
0.02	0.38	0.076	0.006	0.254
0.01	0.19	0.038	0.003	0.127

L_r,t_ presents the overall losses in the receiving and transmitting antennas.

**Table 8:** Received power by the slot antenna as a function of the transmitted power and computed, Vc, and measured, Vmes, harvested voltages.

*F *= 2.45 GHz *R *= 1 kΩ	*R *= 0.2 m, *G_r_* = 3 dB *G_t_* = 6 dB, Matched Antennas			*R *= 0.5 m, *G_r_* = 3 dB, *G_t_* = 6 dB, Matched Antennas		
*P_t_* (mW)	*P_r_* (mW)	Vc volt	Vmes volt	*P_r_* (mW)	V volt	Vmes volt
100	1.9	1.38	1.03	0.302	0.55	0.41
50	0.94	0.97	0.73	0.151	0.388	0.3
20	0.38	0.62	0.47	0.006	0.077	0.06
10	0.19	0.435	0.33	0.003	0.055	0.04

**Table 9:** Comparison of measured harvesting modules’ efficiency as a function of the harvested RF energy.

Harvester	Frequency GHz	Harvested Energy dBm	PCE, Efficiency %	Reference
Metamaterial antenna with strips	0.2–0.4	5–10	65–75	This work
T-shape slot	2–6	10–20	60–75	This work
Octagonal patch	2.4–4.2	0–20	25–80	[51,52,53,54,55,56,57,58]
Metamaterial antenna SRR	2.45	0–10	45–65	[53]
Cross dipole	1.8–2.5	−10	55	[54]

**Table 10:** Simulation and measured results of wearable antennas with energy-harvesting units.

Sensors	Frequency (GHz)	BW %	BW% Measured	Computed Gain dBi	Measured Gain dBi	Length. (cm)	Efficiency %
Metamaterial antenna with strips	0.15–0.5	50	50	5.5	5.7	19.8	90
Circular polarized patch with CSRR [48]	2.4–2.8	15	14	8.3	8	3.6	90
T-shape slot	0.4–6.4	UWB	UWB	3	3	115	95
Circular patch with CSRR [27]	2.5–2.7	9	9	7.5	7.8	3.6	85
Circular patch without CSRR [13,14]	2.6–2.65	1.5	1.8	4.5	4.3	4.8	85
Printed dipole with CSRR [13,14]	0.32–0.36	11	10	5.6	5.7	19.8	95
Dipole without CSRR [13,14]	0.38–0.42	10	12	2.5	2.5	21	90
Stacked circular patch with CSRR [27]	2.6–2.8	9	10	8.5	8.4	4	95
Stacked circular patch without CSRR [27]	2.6–2.8	8	8	5.4	5.3	4.8	89

## Data Availability

Data are unavailable due to privacy.
